# miR-149 Suppresses the Proliferation and Metastasis of Human Gastric Cancer Cells by Targeting FOXC1

**DOI:** 10.1155/2021/1503403

**Published:** 2021-12-17

**Authors:** Dan Li, Yunqing Zhang, Yulong Li, Xiaofei Wang, Fenghui Wang, Juan Du, Huahua Zhang, Haiyan Shi, Yanfeng Wang, Yi Gao, Yun Feng, Jing Yan, Yajuan Xue, Yang Yang, Jing Zhang

**Affiliations:** ^1^Department of Cell Biology and Genetics, Medical College of Yan'an University, Yan'an, 716000 Shaanxi Province, China; ^2^Yan'an Key Laboratory of Chronic Disease Prevention and Research, Yan'an, 716000 Shaanxi Province, China; ^3^Laboratory of Obstetrics and Gynecology, Affiliated Hospital of Yan'an University, Yan'an, 716000 Shaanxi Province, China; ^4^Department of Gastroenterology, Shaanxi Provincial People's Hospital, Xi'an 710068, China; ^5^Department of Cell Biology and Genetics, School of Basic Medical Sciences, Xi'an Jiaotong University Health Science Center, Xi'an, 710061 Shaanxi, China; ^6^Department of Toxicology and Sanitary Analysis, School of Public Health, Xi'an Jiaotong University Health Science Center, Xi'an 710061, China

## Abstract

**Purpose:**

Gastric cancer is one of the most common cancers in the world. miRNAs play an important role in regulating gene expression by binding with 3′-UTR of the target gene. The aim of this study was to investigate the function of miRNA-149 and FOXC1 in gastric cancer. *Patients and Methods*. qRT-PCR was used to detect the expression of miRNA-149 and FOXC1 in gastric cancer tissues and cells. Human gastric cancer cell lines AGS and MKN28 were cultured and transfected with miR-149 overexpression plasmid and its control or FOXC1 siRNA and its control. The MTT, colony formation, flow cytometry, wound healing, transwell, and western blotting were performed to examine the function of miRNA-149 and FOXC1 in the development of gastric cancer. What is more, dual-luciferase assay and western blotting were used to demonstrated the relationship between miRNA-149 and FOXC1.

**Results:**

miRNA-149 was underexpressed in gastric cancer tissues and cells, while overexpression of miRNA-149 promoted cell apoptosis, retarded cell cycle, and inhibited proliferation and migration in AGS and MKN28 cells. In addition, we showed that miRNA-149 targeted FOXC1. What is more, FOXC1 was highly expressed in gastric cancer tissues and cells; the silencing of FOXC1 inhibited the biological function of AGS and MKN28 cells.

**Conclusion:**

miRNA-149 inhibits the biological behavior of gastric cancer by targeting FOXC1, providing a promising target in the treatment of human gastric cancer.

## 1. Introduction

Gastric cancer is a highly recurrent malignancy. The incidence rate of gastric cancer is fifth in the world, and the mortality rate is third, second only to lung cancer and colorectal cancer [1]. The detection rate of early gastric cancer is low and lacks specificity. Therefore, most patients are diagnosed at advanced stages and have a poor overall prognosis [2]. In light of the advancements in tumor biology and molecular technology, further research into the molecular mechanism involved in gastric cancer and identification of biomarkers with high specificity and good targeting [3] is of great significance for the diagnosis and treatment of this condition. MicroRNAs (miRNAs) are noncoding RNAs of approximately 22 nucleotides, which are key regulators of gene expression [4]. Approximately 3% of the human genome codes for miRNA sequences, which can suppress gene expression by mediating translational repression [5]. Recently, the molecular mechanism of miRNAs in tumors has been a popular research topic in tumor-related fields. Several studies have confirmed that miRNA can be used as an oncogene or tumor suppressor gene to regulate the occurrence and development of gastric cancer [6]. Therefore, investigating the expression and regulation of miRNAs may elucidate the mechanism of gastric cancer. miR-149 is a type of miRNA, which can regulate the biological functions of tumors by targeting multiple genes, such as TGF-*β*2, GIT1, and HDAC4 [7–9]. FOXC1 is a member of the FOX superfamily of transcription factors and is located in the cell nucleus. It possesses a unique forkhead domain combined with a fragment of the target gene to initiate transcription [10] and regulates various biological processes such as tumor cell differentiation, cell proliferation, and migration [11]. It is reported that FOXC1 plays an important role in the occurrence, development, and migration of malignant tumors [12]. Studies [13–15] have shown that miR-149 plays an important role in many cancers, although its regulatory role in the FOXC1-mediated progression of gastric cancer remains unclear. In this study, we investigated the inhibitory effects of miR-149 on the biological functions of gastric cancer cells by targeting FOXC1.

## 2. Materials and Methods

### 2.1. Tissues and Cells

Twenty pairs of gastric cancer and adjacent nontumor tissues, which were not subjected to radiotherapy or chemotherapy, were obtained from the First Affiliated Hospital of Xi'an Jiaotong University. Patients provided written informed consent. This study was approved by the ethics committee of Yan'an University School of Medicine (No. YADX2020-139). The human gastric epithelial permanent cell line (GES-1), human gastric cancer cell lines (AGS, BGC-823, MKN45, and SGC-7901), and HEK293 cells were provided by the Biomedical Experiment Center of Xi'an Jiaotong University. MKN28 cells used in our experiment were purchased from Guangzhou Saiku Biotechnology Co., Ltd. (http://www.cellcook.com/h-pd-116.html?fromColId=30), which identified MKN28 cells by STR typing and determined that MKN28 cells were derived from MKN74 cells. The cells were cultured in RPMI-1640 medium or DMEM (Basal media, Shanghai, China) containing 10% fetal bovine serum (FBS, Biological Industries, Israel) in a humidified incubator with 5% CO_2_ at 37°C.

### 2.2. Bioinformatics

Gene expression data of miRNA-149 and FOXC1 in cancer and tumor-adjacent tissues of gastric cancer patients were downloaded from The Cancer Genome Atlas (TCGA) database. The sequence information of human miR-149 was derived from miRBase. Target sequences of miR-149 and FOXC1 were predicted using TargetScan.

### 2.3. qRT-PCR

Total RNA from gastric cancer tissues and cells was extracted using TRIzol reagent (Thermo Fisher Scientific, Waltham, MA, USA). The PrimeScript RT Reagent Kit (GenStar, Beijing, China) was used to synthesize cDNA, which was amplified using the SYBR Premix Ex Taq II Kit (GenStar, Beijing, China) to detect the expression of miR-149 and FOXC1. Primer sequences are listed in Table 1.

### 2.4. Expression Vector Construction and Transfection

The sequence of pre-miR-149 was obtained from the miRbase database, and the binding sequences of FOXC1 and miR-149 were obtained from TargetScan. The miR-149 expression vector was constructed using synthetic oligonucleotides and cloned into the pcDNATM6.2-GW/EmGFP vector between the EcoRI and HindIII sites. The 3′-UTR of FOXC1 was synthesized and cloned into the pmiR-GLO vector between the SacI and XhoI sites. Small interfering RNAs (siRNAs) targeting FOXC1 and NC were purchased from GenePharma Biotech (Shanghai, China). The sequences are presented in Table 1. Vector or siRNA transfection was performed according to standard protocols using PolyPlus (PolyPlus, Illkirch-Graffenstaden, France).

### 2.5. MTT Assay

Cells in the logarithmic growth phase were seeded in a 96-well plate. Each group comprised three wells with each well containing 100 *μ*L of culture medium. The cells were cultured for 24 h and then transfected. The action of succinate dehydrogenase on mitochondria of living cells changes the light-yellow color of the solution to blue-purple or blue-black upon the formation of MTT-formazan. The cells were collected at 24, 48, and 72 h, and 10 *μ*L MTT was added to each well. After incubation at 37°C for 4 h, the culture medium was discarded, 150 *μ*L dimethyl sulfoxide (DMSO) was added, and the optical density was measured at a wavelength of 490 nm using a FLUOstar Omega microplate reader (BMG Labtech, Offenburg, Germany).

### 2.6. Colony-Formation Assay

Viable cells were cultured in 6-well plates for 24 h. After 24 h of transfection, cells were seeded into a 6-well plate at a density of 1 × 10^3^ cells/well. After 1-2 weeks of culture to allow for colony formation, the cells were fixed with 4% paraformaldehyde for 15 min and stained with 0.1% crystal violet for 30 min. Colonies were washed with phosphate-buffered saline (PBS) and imaged. Thereafter, DMSO was added and the absorbance was measured at 570 nm using a FLUOstar Omega microplate reader.

### 2.7. Cell Cycle Analysis

Cells in the logarithmic growth phase were cultured in 12-well plates for 24 h and transfected with synchronized cells in a serum-free medium. After 24 h, the cells were digested and fixed with 1 mL of precooled 70% ethanol and washed twice with PBS. Next, the cells were incubated with 150 *μ*L of RNase for 5 min in the dark and 150 *μ*L of PI on ice for 30 min and analyzed using flow cytometry.

### 2.8. Cell Apoptosis Assay

Cells in the logarithmic growth phase were inoculated into 12-well plates. After 24 h of culture, the cells were transfected when the degree of fusion was >60%. After 48 h, an Annexin-V FITC cell apoptosis detection kit was used to stain the cells and the apoptotic rate was examined using flow cytometry.

### 2.9. Wound Healing Assay

Three straight lines were drawn from the bottom of a 6-well plate using a marking pen. Cells with a good logarithmic growth phase were seeded in a well plate for 24 h. When the degree of fusion of the cells reached 80–90%, the cells were transfected. PBS was used to wash off the exfoliated cells and collect images for 0 h. Thereafter, 2 mL of medium containing 1% FBS was added to the plates for further culture, and images were captured at 24 and 48 h.

### 2.10. Transwell Assay

Cells were cultured in a 12-well plate and transfected when the fusion degree of cells reached 60–70%. After 24 h, the cells were collected, followed by digestion and centrifugation. Approximately 200 *μ*L of the cell suspension (2 × 10^5^ cells without FBS) was added into the upper chamber of a transwell chamber (Millipore, Sigma), and 600 *μ*L of medium containing 10% FBS was added into the lower chamber of the transwell chamber. After 24 h of incubation, the cells were fixed with 4% paraformaldehyde for 15 min and stained using 1% crystal violet for 30 min. The cells that did not pass through the membrane were removed, and the images captured using microscopy. Lastly, 600 *μ*L of DMSO was added to the chamber and the absorbance was measured at 570 nm.

### 2.11. Dual-Luciferase Assay

HEK-293 cells were cultured in 96 well plates (4 × 10^3^ cells/well). After 24 h, the miR-149 expression vector was cotransfected with FOXC1-WT or FOXC1-MT vectors into HEK-293 cells, and the pmiR-GLO vector was cotransfected as a control. After 24 h of transfection, the luciferase activity of each group was detected using a dual-luciferase reporter assay system (Promega, Madison, WI, USA).

### 2.12. Western Blotting

After transfection for 48 h, the total protein was extracted and the protein concentration was determined using a BCA protein assay kit (GenStar, Beijing, China). The proteins were separated using 10% sodium dodecyl sulfate-polyacrylamide gel electrophoresis and transferred to a polyvinylidene fluoride membrane using electrophoretic transfer (Millipore, MA, USA). After blocking with 5% skimmed milk at room temperature for 1 h, the membranes were incubated with rabbit anti-human FOXC1 (1 : 1000, Abcam, Cambridge, UK; Rabbit MAb-ab226219), Bax (1 : 5000, Proteintech, Wuhan, China, 50599-2-Ig), Bcl2 (1 : 1000, Proteintech, Wuhan, China, 12789-1-AP), CDK4 (1 : 1000, Proteintech, Wuhan, China, 11026-1-AP), MMP2 (1 : 1000, Proteintech, Wuhan, China, 10373-2-AP), MMP9 (1 : 1000, Proteintech, Wuhan, China, 27306-1-AP), E-cadherin (1 : 1000, Proteintech, Wuhan, China, 20874-1-AP), and *β*-actin (1 : 5000, Abways, Shanghai, China, AB0035) at 4°C overnight. After washing the membrane three times with TBST, the membrane was incubated with a secondary antibody (1 : 5000, Abways, Shanghai, China, AB0101) for 1 h. Then, the membranes were washed three times with TBST and incubated with ECL Plus Reagent (Millipore, USA). Lastly, the blots were detected using a supersensitive enhanced chemiluminescence system (Pierce, IL, USA).

### 2.13. Immunohistochemistry

Tissues were fixed in 10% formalin to prepare paraffin sections (5 *μ*m thick). The sections were deparaffinized with xylene and hydrated using gradient alcohol, followed by antigen retrieval and nonspecific antigen blocking. After incubation with the primary antibody (FOXC1, 1 : 50, Abcam, Cambridge, UK, Rabbit MAb-ab226219) and the secondary antibody, the slides were dehydrated and stained with 3,3-diaminobenzidine (DAB) and hematoxylin. The sections were observed using a stereomicroscope, and digital images were captured and analyzed using a Leica image analysis system.

### 2.14. Statistical Analysis

Data are presented as mean ± standard deviation and analyzed using SPSS version 22.0. Student's *t*-test was used to compare the differences between the treatment and control groups. *p* < 0.05 was considered statistically significant.

## 3. Results

### 3.1. miR-149 Is Downregulated in Gastric Cancer Tissues and Cells

**Figure 1:**
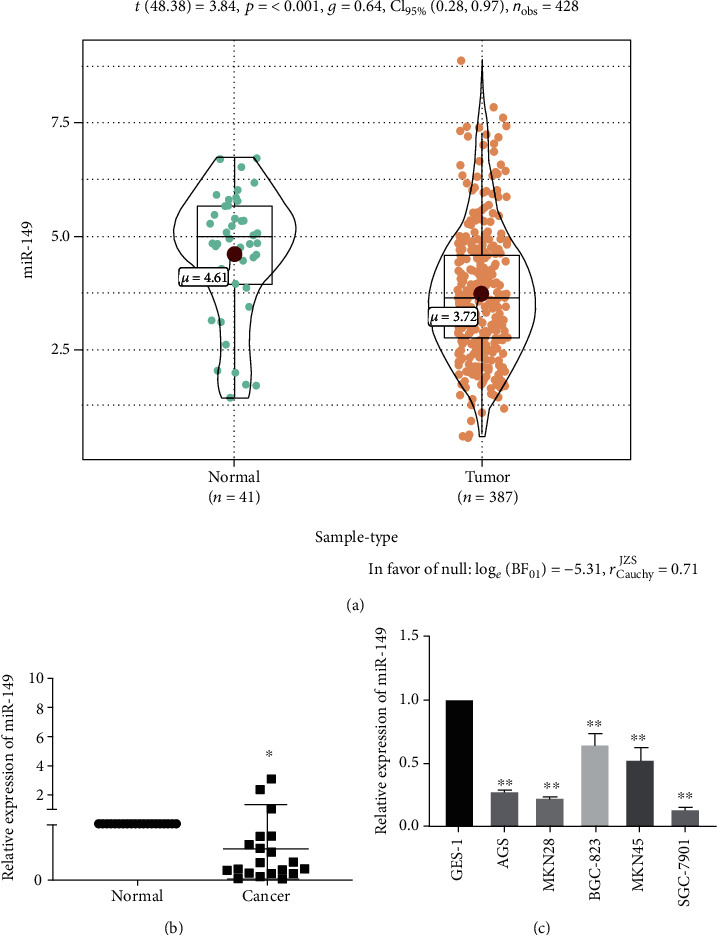
miR-149 is downregulated in gastric cancer (GC) tissues and cell lines. (a) Expression analysis of miR-149 expression in GC tissues (*n* = 387) and normal tissues (*n* = 41) was performed in TCGA database. (b) qRT-PCR showed that miR-149 expression significantly decreased in GC tissues compared with that in normal gastric tissues (*n* = 20). (c) qRT-PCR was performed to analyze the miR-149 expression in GC cell lines (AGS MKN28, BGC-823, MKN45, and SGC-7901)) and a normal gastric epithelial cell line (GES-1). Differences between groups were evaluated using Student's *t*-test, and data are expressed as mean ± SD. For all data, ∗ indicates *p* < 0.05, and ∗∗ indicates *p* < 0.01.

We analyzed the expression of miR-149 in gastric cancer tissues from TCGA database, and the results showed that the expression level in 387 gastric cancer tissues was significantly lower than that in 41 nontumor gastric cancer tissues (*p* < 0.01) (Figure 1(a)). To verify these results, we performed qRT-PCR to detect the expression of miR-149 in 20 pairs of gastric cancer tissues and adjacent normal tissues. The results showed that the expression level of miR-149 was lower in gastric cancer tissues than that in adjacent tissues (Figure 1(b)). Additionally, we compared the expression of miR-149 between different cell lines, and the results showed that miR-149 expression was lower in five gastric cancer cell lines than that in GES-1 cells (Figure 1(c)).

### 3.2. Overexpression of miR-149 Inhibits the Proliferation and Metastasis of Gastric Cancer AGS and MKN28 Cells

**Figure 2:**
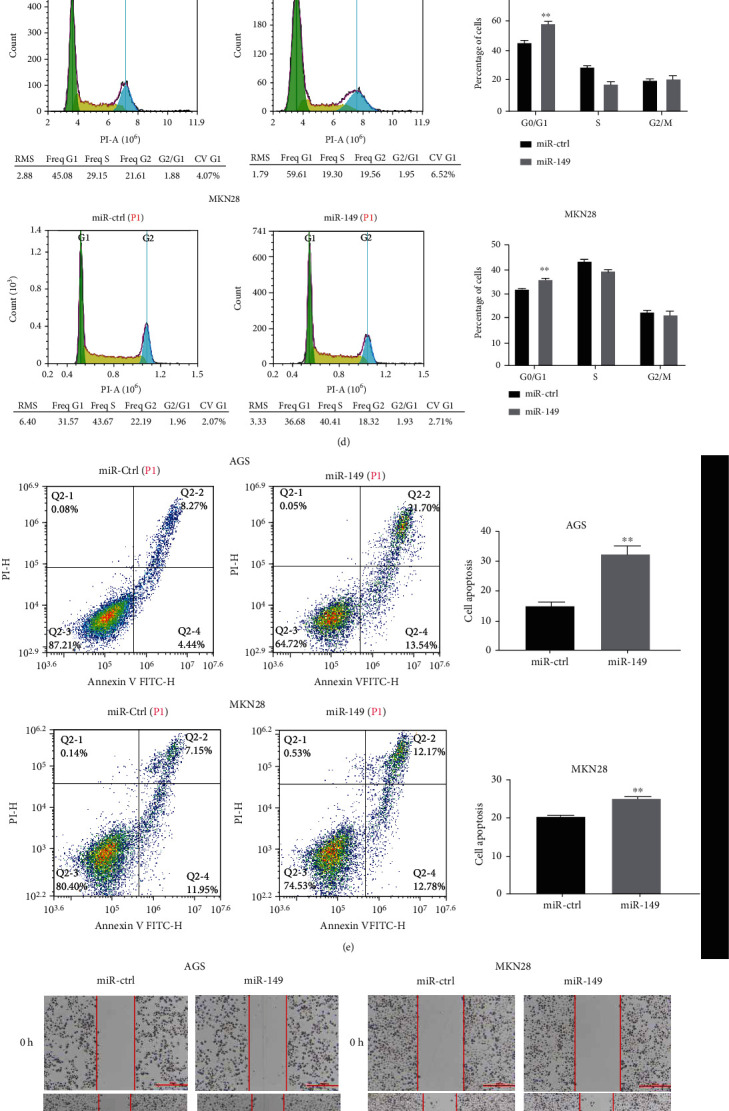
miR-149 overexpression repressed the proliferation and metastasis of gastric cancer cells. AGS and MKN28 cells were transfected with miR-149 overexpression plasmid or miR-ctrl plasmid. (a) miR-149 expression was examined by qRT-PCR assay after transfection. (b) Cell proliferation was detected using the MTT assay at 24, 48, and 72 h. (c) Colony formation was assessed in AGS/MKN28 cells after transfection. (d) Flow cytometry analysis of cell cycle in AGS and MKN28 cells. (e) Flow cytometry analysis of apoptosis after transfection with miR-149 overexpression plasmid or miR-ctrl plasmid. (f, g) Wound-healing and transwell assays showed that miR-149 suppressed GC cell migration and invasion abilities. (h) Western blot to detect the expression of Bax, Bcl2, CDK4, MMP2, MMP9, and E-cadherin after transfection with miR-149 overexpression plasmid or miR-ctrl plasmid in AGS and MKN28 cells. The difference between the two groups was evaluated by Student's *t*-test, and data are expressed as mean ± SD. For all data, ∗ means *p* < 0.05, and ∗∗ means *p* < 0.01. Differences between groups were evaluated using Student's *t*-test, and data are expressed as mean ± SD. For all data, ∗ indicates *p* < 0.05, and ∗∗ indicates *p* < 0.01.

To investigate the role of miR-149 in gastric cancer cells, we successfully constructed an miR-149 overexpression vector and transfected it into AGS and MKN28 cells. The results of qRT-PCR showed that the expression of miR-149 was significantly increased after transfection in the miR-149-overexpression group (*p* < 0.01) (Figure 2(a)). MTT and clone formation experiments showed that the overexpression of miR-149 significantly inhibited cell proliferation and clone number in AGS and MKN28 cells (Figures 2(b) and 2(c)). Flow cytometry revealed that miR-149 blocked the cell cycle in the G0/G1 phase (Figure 2(d)). Annexin V and PI double staining was used to observe the effect of miR-149 on gastric cancer cell apoptosis. Overexpression of miR-149 induced apoptosis (Figure 2(e)), whereas the results of the wound-healing and transwell assays indicated that miR-149 inhibited the migration and invasion of AGS and MKN28 cells, (Figures 2(f) and 2(g)). In order to explore the mechanism of miR-149 in the cell cycle, apoptosis, and migration of gastric cancer, we detected the expression levels of cell cycle, apoptosis, and migration-related proteins. The transfection of AGS and MKN28 cells with miR-149 downregulated the expression of CDK4 and Bcl2 and upregulated the expression Bax. Overexpression of miR-149 upregulated the expression of E-cadherin and downregulated the expression of MMP2 and MMP9 (Figure 2(h)). These results indicate that miR-335-5p is involved in the progression and migration of GCs.

### 3.3. FOXC1 Is a Downstream Gene Associated with miR-149

**Figure 3:**
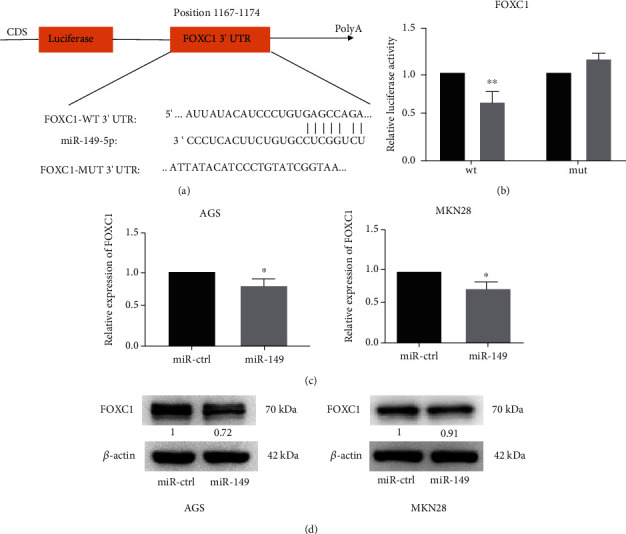
FOXC1 is a direct target of miR-149. (a) miR-149 has the binding sites of the 3′-UTR of FOXC1. (b) Luciferase activity in the pmiRGLO-FOXC1-wt group showed a statistically significant decrease following overexpression of miR-149. (c, d) The mRNA and protein expression levels of FOXC1 were detected using qRT-PCR and western blot after transfection with miR-149 overexpression plasmid or miR-ctrl plasmid in AGS and MKN28 cells. Differences between groups were evaluated using Student's *t*-test, and data are expressed as mean ± SD. For all data, ∗ indicates *p* < 0.05, and ∗∗ indicates *p* < 0.01.

To further investigate the mechanism of miR-149 in regulating the biological behavior of gastric cancer cells, we predicted that miR-149 had binding sites with FOXC1 3′-UTR through the TargetScan database (Figure 3(a)) and constructed FOXC1 wild-type and mutant 3′-UTR luciferase vectors. The miR-149-over and reporter or pmiR-GLO plasmid were cotransfected into HEK-293 cells. The results of the luciferase reporter test showed that the overexpression of miR-149 significantly inhibited the wild-type luciferase activity of FOXC1 (*p* < 0.01) compared to the control or mutant groups (Figure 3(b)). Simultaneously, the results of qRT-PCR and western blotting indicated that the overexpression of miR-149 inhibited the expression of FOXC1 at the mRNA and protein levels in SGC-7901 and MKN28 cells compared to that in the control group (Figures 3(c) and 3(d)). These findings indicated that miR-149 could target FOXC1.

### 3.4. FOXC1 Is Highly Expressed in Gastric Cancer Tissues and Cells

**Figure 4:**
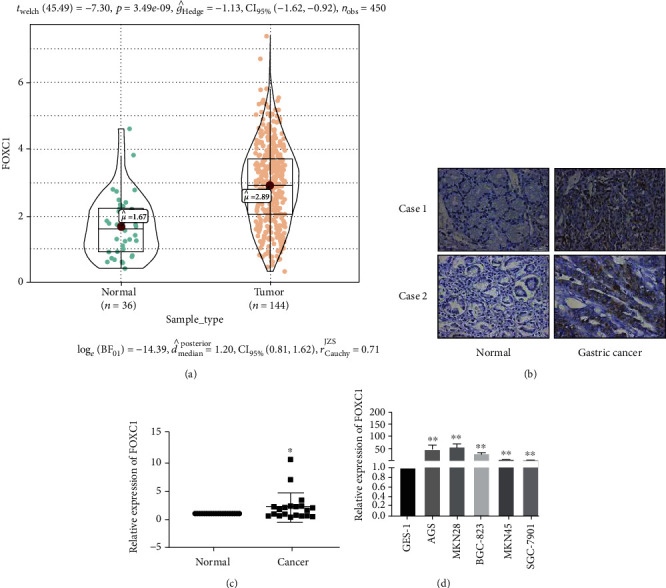
FOXC1 is upregulated in gastric cancer. (a) Expression analysis of FOXC1 expression in GC tissues (*n* = 414) and normal tissues (*n* = 36) was performed in TCGA database. (b, c) immunohistochemistry and qRT-PCR showed that FOXC1 expression significantly increased in GC tissues compared with that in normal gastric tissues (*n* = 20). (d) qRT-PCR was performed to analyze the FOXC1 expression in GC cell lines (AGS MKN28, BGC-823, MKN45, and SGC-7901) and GES-1 cells. Differences between groups were evaluated using Student's *t*-test, and data are expressed as mean ± SD. For all data, ∗ indicates *p* < 0.05, and ∗∗ indicates *p* < 0.01.

Analysis of the expression of FOXC1 in gastric cancer tissues using TCGA database showed that it was highly expressed in 414 gastric cancer tissues compared to that in 36 adjacent tissues (Figure 4(a)). Results from immunohistochemistry and qRT-PCR showed that FOXC1 was highly expressed in gastric cancer tissues (Figures 4(b) and 4(c)). The mRNA levels of FOXC1 suggested that it is highly expressed in gastric cancer cells (Figure 4(d)).

### 3.5. Silencing FOXC1 Inhibits the Proliferation and Metastasis of Gastric Cancer AGS and MKN28 Cells

**Figure 5:**
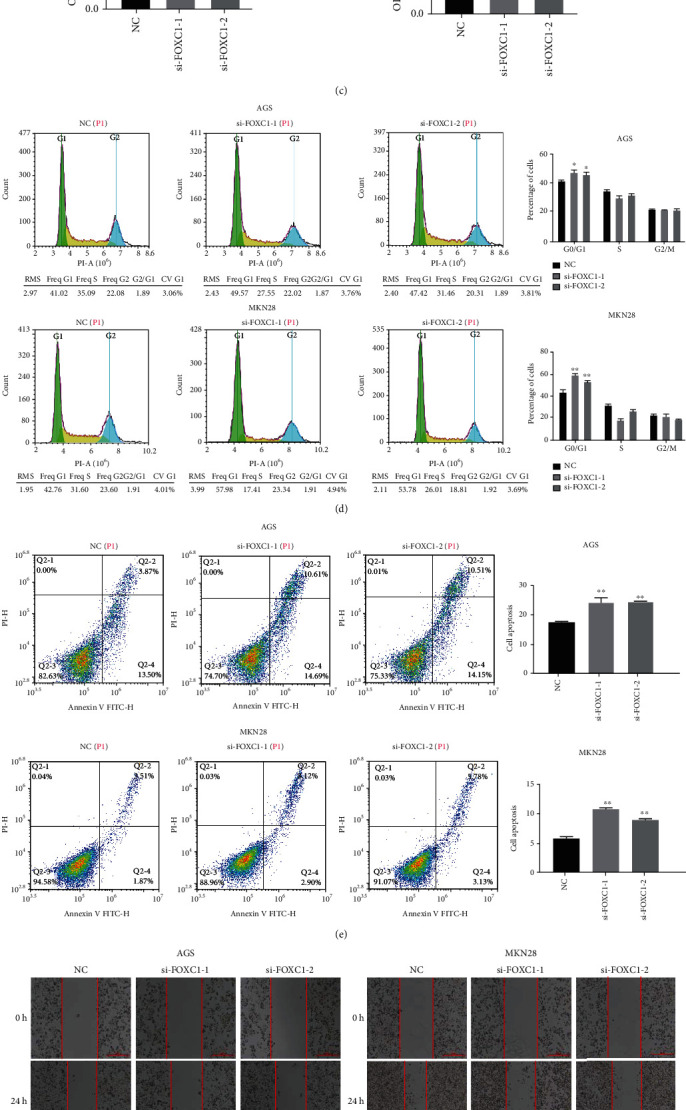
Silencing FOXC1 suppresses cell proliferation and metastasis of gastric cancer cells. AGS cells and MKN28 cells were transfected with si-FOXC1-1, si-FOXC1-2, or a negative control. (a) qRT-PCR and western blot were performed to determine the expression of FOXC1 after transfection. (b) Cell proliferation was detected using the MTT assay after transfection. (c) Colony formation was assessed in AGS/MKN28 cells after transfection. (d) Flow cytometry analysis of cell cycle in AGS and MKN28 cells. (e) Flow cytometry analysis of apoptosis after transfection with si-FOXC1-1, si-FOXC1-2, or the negative control. (f, g) Wound-healing and transwell analysis representing the migration and metastatic capacity of AGS and MKN28 cells. (h) Western blot to detect the expression of Bax, Bcl2, CDK4, MMP2, MMP9, and E-cadherin after transfection with si-FOXC1-1, si-FOXC1-2, or a negative control. Differences between groups were evaluated using Student's *t*-test, and data are expressed as mean ± SD. For all data, ∗ indicates *p* < 0.05, and ∗∗ indicates *p* < 0.01.

To further investigate the function of FOXC1 in gastric cancer cells, we used RNA interference technology to silence the expression of FOXC1 in AGS and MKN28 cells. Results from qRT-PCR and western blotting showed that after transfection of si-FOXC1 in AGS and MKN28 cells, the mRNA and protein expression of FOXC1 decreased significantly (*p* < 0.01) (Figure 5(a)). MTT and clone formation experiments showed that silencing FOXC1 significantly inhibited the cell viability and clone number of AGS and MKN28 cells (Figures 5(b) and 5(c)). Results from flow cytometry showed that transfection with interfering RNA si-FOXC1 blocked the cell cycle in the G0/G1 phase (Figure 5(d)). Annexin V and PI double staining revealed that compared to the control group, silencing FOXC1 induced apoptosis of gastric cancer cells (Figure 5(e)). Wound-healing and transwell assays showed that FOXC1 silencing inhibited the migration and invasion of AGS and MKN28 cells (Figures 5(f) and 5(g)). Based on a western blot analysis, silencing FOXC1 decreased the expression levels of Bcl2, CDK4, MMP2, and MMP9 and increased Bax and E-cadherin expression (Figure 5(h)).

## 4. Discussion

The occurrence of gastric cancer is a multistage and multifactorial pathological process involving gene mutations, epigenetic changes, and abnormal molecular signaling pathways [16]. miRNAs affect the stability and translation of target mRNAs and regulate processes such as cell cycle [17], proliferation [18], apoptosis [19], migration [20], and invasion [21]. Recently, numerous studies have shown that abnormal expression of miRNAs is closely related to the occurrence, development, and metastasis of gastric cancer. It has been reported that miR-96-5p is downregulated in gastric cancer tissues and inhibits the proliferation, migration, and epithelial-mesenchymal transition of gastric cancer cells by targeting FOXQ1 [22]. High expression of miR-103a-3p in gastric cancer tissue promotes cell growth causing the cell cycle to transition from the S phase to G_2_/M phase and plays the role of an oncogene [23]. miR-34a is downregulated in gastric cancer and targets PDGFR and MET through the PI3K/Akt pathway to inhibit tumorigenesis of gastric cancer [24]. miR-149 is abnormally expressed in several malignant tumors and can be used as an oncogene or tumor suppressor gene to regulate the biological behavior of tumor cells. miR-149 inhibits the proliferation and migration of hepatocellular carcinoma cells and hepatocarcinogenesis by impeding the NF-*κ*B and STAT3 signaling pathways [14]. Low expression of miR-149 in colon cancer inhibits the migration and invasion of cells and is associated with lymph node or distant metastasis and TNM stage [25]. However, the expression level of miR-149 is increased in prostate cancer, which can enhance motility and invasiveness of cancer cells by downregulating DAB2IP and promote activation of NF-*κ*B signaling and expression of proangiogenic factors to enhance tumor progression [26]. Low expression of miR-149 in gastric cancer was detected by analyzing TCGA database and confirmed using qRT-PCR in 20 pairs of gastric cancer tissues and five gastric cancer cell lines, suggesting that miR-149 plays a role as a tumor suppressor gene in gastric cancer. Moreover, miR-149 inhibited the growth and migration of gastric cancer cells, arrested the cell cycle in the G0/G1 phase, and promoted apoptosis. Overexpression of miR-149 resulted in upregulated expression of Bax, downregulated expression of Bcl-2, downregulated expression of CDK4, upregulated expression of E-cadherin, and downregulated expression of MMP2 and MMP9. The results suggest that miR-149 is a tumor suppressor affecting the biological function of gastric cancer cells. One research found that miR-149 exerts its role of inhibiting GC cell proliferation and cell cycle progression by targeting ZBTB2 thereby modulating the expression of downstream regulators of cell cycle progression [27]. However, each miRNA may regulate many target genes affecting tumorigenesis in different ways, and different target genes have different effects on tumor cell function. This study is done to investigate whether FOXC1 as a target gene of miR-149 mediates its inhibitory effect on gastric cancer cells. FOXC1 is an important molecule in the regulation of mesoderm, neural crest, and eye development. It was first found to be associated with Axenfeld-Rieger syndrome, with mutations in patients with Rieger's abnormality, iris dysplasia, and Axenfeld abnormality [28], and its mutation typically causes glaucoma or eye hypoplasia. Recently, an increasing number of studies have shown that the expression of FOXC1 in many human malignant tumors results in a change in the expression of a series of target genes, which regulate the proliferation, migration, and invasion of tumor cells in colorectal cancer. FOXC1 is overexpressed and promotes colorectal cancer metastasis by activating the expression of ITGA7 and FGFR4 [29]. FOXC1 is upregulated in non-small-cell lung cancer tissues and is negatively correlated with patient survival. Additionally, it can enhance the properties of tumor stem cells by targeting *β*-catenin directly [30]. FOXC1 plays an important role in the development of pancreatic cancer as it promotes metastasis by enhancing cell proliferation, migration, invasion, epithelial-mesenchymal transition, and angiogenesis [31]. The expression of FOXC1 is regulated by multiple factors including DNA methylation and histone modification [32], regulation of miRNA and long noncoding RNA [33, 34], and phosphorylation and ubiquitination modification [35]. Numerous studies have shown that FOXC1 can participate in the occurrence and development of tumors as a target gene of multiple miRNAs. In cervical cancer, overexpression of miR-374c-5p can inhibit the expression of FOXC1 [36]. Additionally, miR-138-5p can directly target FOXC1 to regulate the invasion and migration of cervical cancer [37]. In throat cancer, miR-204-5p has a negative regulatory effect on the expression of FOXC1 [38]. However, the ability of miR-149 to regulate the biological functions of gastric cancer cells by targeting FOXC1 remains unclear. miR-149 and FOXC1 3′-UTR have complementary nucleotide sequences based on bioinformatics predictions. The dual-fluorescein reporter gene experiment confirmed that FOXC1 is a miR-149 target gene, and overexpression of miR-149 can inhibit FOXC1 mRNA and protein expression. The expression of FOXC1 was upregulated in gastric cancer tissues and cell lines compared to that in control nontumor tissues and normal gastric epithelial cell lines, which was consistent with our prediction from TCGA database. Additionally, we elucidated the effect of FOXC1 on the function of gastric cancer cells. Silencing FOXC1 significantly inhibits the growth and migration of gastric cancer AGS and MKN28 cells, promotes apoptosis thereof, and blocks the cell cycle in the G1 phase. After silencing FOXC1, the expression of Bcl-2, CDK4, MMP2, and MMP9 was downregulated, and Bax and E-cadherin were upregulated. These results suggest that FOXC1 function as an oncogene in gastric cancer cells. Silencing FOXC1 was consistent with overexpression of miR-149 on the proliferation, cycle, apoptosis, and migration of gastric cancer cells, which suggest that FOXC1 mediates the regulation of miR-149 in gastric cancer progression.

## 5. Conclusion

miR-149 is low expressed in gastric cancer cells and inhibits the proliferation and metastasis of gastric cancer AGS and MKN28 cells by targeting FOXC1, which may be a potential new target for GC therapy.

## Figures and Tables

**Table 1:** Primers and oligonucleotides used in this work.

Name	Sequence (5′–3′)
miR-149-RT	GTCGTATCCAGTGCGTGTCGTGGAGTCGGCAA TTGCACTGG ATACGACGGGAGTG
miR-149-F	ATCCAGTGCGTGTCGTG
miR-149-R	TGCTTCTGGCTCCGTGTCTT
U6-RT	CGCTTCACGAATTTGCGTGTCAT
U6- F	GCTTCGGCAGCACATATACTAAAAT
U6-R	CGCTTCACGAATTTGCGTGTCAT
FOXC1-F	TGTTCGAGTCACAGAGGATCG
FOXC1-R	ACAGTCGTAGACGAAAGCTCC
GAPDH-F	GTAGAGGCAGGGATGATGTTC
GAPDH-R	GCCAAAAGGGTCATCATCTC
Pre-miR-149	GCCGGCGCCCGAGCUCUGGCUCCGUGUCUUC ACUCCCGUGCUUGUCCGAGGAGGGAGGGAGGG ACGGGGGCUGUGCUGGGGCAGCUGGA
FOXC1-3′UTR	AUUAUACAUCCCUGUGAGCCAGA
miR-149-sense	AATTCGCCGGCGCCCGAGCTCTGGCTCCGTGTCTT CACTCCCGTGCTTGTCCGAGGAGGGAGGGAGGGA CGGGGGCTGTGCTGGGGCAGCTGGAA
miR-149-antisense	AGCTTTCCAGCTGCCCCAGCACAGCCCCCGTCCCTC CCTCCCTCCTCGGACAAGCACGGGAGTGAAGACACG GAGCCAGAGCTCGGGCGCCGGCG
FOXC1-3′UTR-WT	CATTATACATCCCTGTGAGCCAGAC
FOXC1-3′UTR-WB	TCGAGTCTGGCTCACAGGGATGTATAATGAGCT
FOXC1-3′UTR-MT	CATTATACATCCCTGTATCGGTAAC
FOXC1-3′UTR-MB	TCGAGTTACCGATACAGGGATGTATAATGAGCT
NC-sense	UUCUCCGAACGUGUCACGUTT
NC-antisense	ACGUGACACGUUCGGAGAATT
si-FOXC1-1-sense	CGGGAAUAGUAGCUGUCAATT
si-FOXC1-1-antisense	UUGACAGCUACUAUUCCCGTT
si-FOXC1-2-sense	ACAAGAAGAUCACCCUGAATT
si-FOXC1-2-antisense	UUCAGGGUGAUCUUCUUGUTT

## Data Availability

The data used to support the findings of this study are included within the article.
